# Impact of preoperative nutritional status on postoperative dysphagia after elective cardiovascular surgery in older adults

**DOI:** 10.1111/ggi.14810

**Published:** 2024-01-14

**Authors:** Midori Miyagi, Shinnosuke Okuma, Yoshio Nunoi, Kota Kawada, Takeshiro Fujii, Satoru Ebihara

**Affiliations:** ^1^ Department of Internal Medicine and Rehabilitation Science Tohoku University Graduate School of Medicine Sendai Japan; ^2^ Division of Cardiovascular Surgery, Department of Surgery School of Medicine, Toho University Faculty of Medicine Tokyo Japan

Dear Editor,

Dysphagia following elective cardiovascular surgery causes pneumonia and affects postoperative outcomes and hospital discharge duration. Previously observed factors causing postoperative dysphagia in cardiovascular surgery include intraoperative use of transesophageal echocardiography, placement of artificial heart lungs, and prolonged postoperative intubation.^1^ In recent years, owing to the increasing population of older adults who have undergone cardiovascular surgery, attention has focused on the link between postoperative outcomes and preoperative nutritional status.^2^, ^3^ However, the association between postoperative dysphagia and preoperative nutritional status remains unclear. Thus, this study aimed to elucidate the relationship between preoperative nutritional status, measured using indexes such as the Controlling Nutritional Status (CONUT) score^4^ and Geriatric Nutritional Risk Index (GNRI),^5^ and the incidence of postoperative dysphagia in patients who underwent elective cardiovascular surgery.

We retrospectively reviewed the medical charts of 184 patients over 65 years old who underwent elective open‐heart cardiovascular surgery at the Toho University Omori Medical Center between April 2018 and December 2021. The flowchart of patient selection is shown in Figure S1.. Clinical data collected from patients included demographic information and body mass index (BMI), operation time, length of intensive care unit (ICU) stay, duration of intubation days, type of operation, and presence or absence of recurrent nerve paralysis. BMI was calculated from preoperative patient data. Patients with obvious cerebral infarction (*n* = 4) or in a confused state (Richmond Agitation‐Sedation Scale below −2 or above +2) (*n* = 2) throughout the perioperative period were excluded from this study.

Severity of dysphagia was assessed using Functional Oral Intake Scale (FOIS) scores, which were determined from medical chart descriptions of patients' food intake status^6^ within 2 weeks after surgery. In our hospital, the type of meal given to patients with dysphagia is determined by a meal round that is discussed and decided on by a dentist, speech therapist, specialist nurse for dysphagia, rehabilitation medical doctor, and otolaryngologist. The reference for determining the type of meal is the best ability of a patient with dysphagia. The preoperative FOIS score for all patients in this study was 7. According to the FOIS level, patients were classified into non‐dysphagia (FOIS level: 6–7) and dysphagia (FOIS level: 2–5) groups.^7^ Patients with level 1 postoperative FOIS were excluded owing to complex complications (*n* = 24). The patients with FOIS level 1 had varying conditions, with postoperative complications such as tracheostomy (*n* = 10), intubation (*n* = 5), non‐invasive positive‐pressure ventilation (*n* = 2), continuous hemodiafiltration (*n* = 2), progress of dementia (*n* = 1), septic shock (*n* = 1), delirium (*n* = 1), acute respiratory distress syndrome (*n* = 1), and paraplegia (*n* = 1).

GNRI was calculated using the following formula:^5^

$$\text{GNRI}=\left[14.89\times \mathrm{Alb}\;\text{level}\;\left(g/L\right)\right]+\left[41.7\times \left(\text{actual weight}/\text{reference weight}\right)\right].$$

The CONUT score was calculated based on serum albumin levels, total lymphocyte counts, and total cholesterol levels.^4^ Preoperative blood samples were obtained within two weeks of surgery.

In total, 154 older patients underwent elective cardiovascular surgery. Based on FOIS scores, 13 patients (8.4%) experienced dysphagia after elective cardiovascular surgery. Comparisons of perioperative clinical data from the medical charts between the non‐dysphagia and dysphagia groups are shown in Table 1. In the dysphagia group, BMI was significantly lower, and ICU stay and intubation days were significantly longer. Comparing nutrition status data for all participants, the GNRI and CONUT scores were 97.5 ± 7.4 and 2.07 ± 2.0, respectively. Comparisons of nutritional status between the non‐dysphagia and dysphagia groups are shown in Table 1. GNRI scores were significantly lower in the dysphagia group than in the non‐dysphagia group. Additionally, multivariate logistic analyses were performed using intubation days, ICU stay, BMI, GNRI and CONUT score as covariates. Here, intubation days (odds ratio [OR] 1.81, 95% confidence interval [CI] 1.24–2.64, *P* = 0.002) and GNRI (OR 0.84, 95% CI 0.72–0.98, *P* = 0.029) remained independent risk factors related to the incidence of dysphagia.

**Table 1:** Comparison of perioperative clinical data

	Non‐dysphagia	Dysphagia	*P*‐value
	*n* = 141	*n* = 13	
Age (year)	74.3 ± 5.2	74.1 ± 5.2	ns^†^
Female/Male	45/96	6/7	ns^‡^
BMI (kg/m^2^)	22.9 ± 3.2	20.9 ± 3.0	0.024
Smoking (*n*)	85	9	ns^§^
Operation time (min)	418.2 ± 114	445.4 ± 156	ns^†^
ICU stay (day)	4.0 ± 2.6	6.8 ± 2.5	*P* < 0.01^†^
Intubation days (day)	1.5 ± 1.3	4.2 ± 3.1	*P* < 0.01^†^
Recurrent nerve paralysis (*n*)	0	3	*P* < 0.01^†^
Type of surgery			
CABG (*n*)	19	2	ns^‡^
Valve (*n*)	81	5	ns^§^
Aorta (*n*)	37	6	ns^‡^
Other (*n*)	4	0	ns^‡^
Concomitant (*n*)	24	3	ns^‡^
Comorbidity			
Hypertension (*n*)	107	9	ns^§^
Diabetes mellitus (*n*)	38	5	ns^§^
GNRI (continuous)	97.8 ± 7.4	93.5 ± 6.2	0.024^†^
CONUT (continuous)	2.0 ± 2.0	2.5 ± 1.3	ns^†^

Values are mean ± standard deviation or number. BMI, body mass index; CABG, coronary artery bypass grafting.

^†^ Mann–Whitney *U* test.

^‡^ Fisher's exact test.

^§^ χ^2^ test.

We found that the preoperative GNRI was significantly lower in the dysphagia group; further, it was an independent prognostic factor for the incidence of dysphagia in patients undergoing elective cardiovascular surgery. Bouillanne *et al*. noted that GNRI, derived from serum albumin and body weight, is reportedly an efficient tool for assessing nutritional status and is associated with mortality and morbidity in hospitalized older patients.^5^ Additionally, it has been used as a predictor of cardiac disease. Another report showed that in patients who underwent elective cardiovascular surgery, a GNRI < 91 was associated with increased risk of mortality.^8^ In older patients with heart failure, low GNRI predicted all‐cause mortality, and GNRI at 2 weeks after admission may be a predictor for discharge to home.^9^ These findings are consistent with our results. Thus, GNRI may be appropriate for a short‐term, unchanging preoperative assessment of cardiac surgery.

The present study has some limitations. This study involved a single‐center design, and swallowing assessment was performed only using the FOIS.

In conclusion, preoperative GNRI can possibly be a predictor of the incidence of postoperative dysphagia. Therefore, preoperative nutritional status should be considered in older patients undergoing elective cardiovascular surgery.

## Disclosure statement

The authors declare no conflict of interest.

## Supporting information


**Supplementary Figure S1.** Flowchart of patient selection. We included patients older than 65 years who underwent elective cardiovascular surgery between April 2018 and December 2021 and excluded patients with obvious cerebral infarction, in a confused state, and with level 1 on the postoperative Functional Oral Intake Scale (FOIS).

## Data Availability

The data supporting the findings of this study are available from the corresponding author upon reasonable request.
