# Supplementary material for: Impact of preoperative nutritional status on postoperative dysphagia after elective cardiovascular surgery in older adults
Source: Geriatr Gerontol Int. 2024 Jan 14;24(2):242–4. doi: 10.1111/ggi.14810 (PMC11503536; doi:10.1111/ggi.14810)
Supplement: Supplementary file 1 — Supplementary Figure S1. Flowchart of patient selection. We included patients older than 65 years who underwent elective cardiovascular surgery between April 2018 and December 2021 and excluded patients with obvious cerebral infarction, in a confused state, and with level 1 on the postoperative Functional Oral Intake Scale (FOIS). [file GGI-24-242-s001.pptx]

## Slide 1
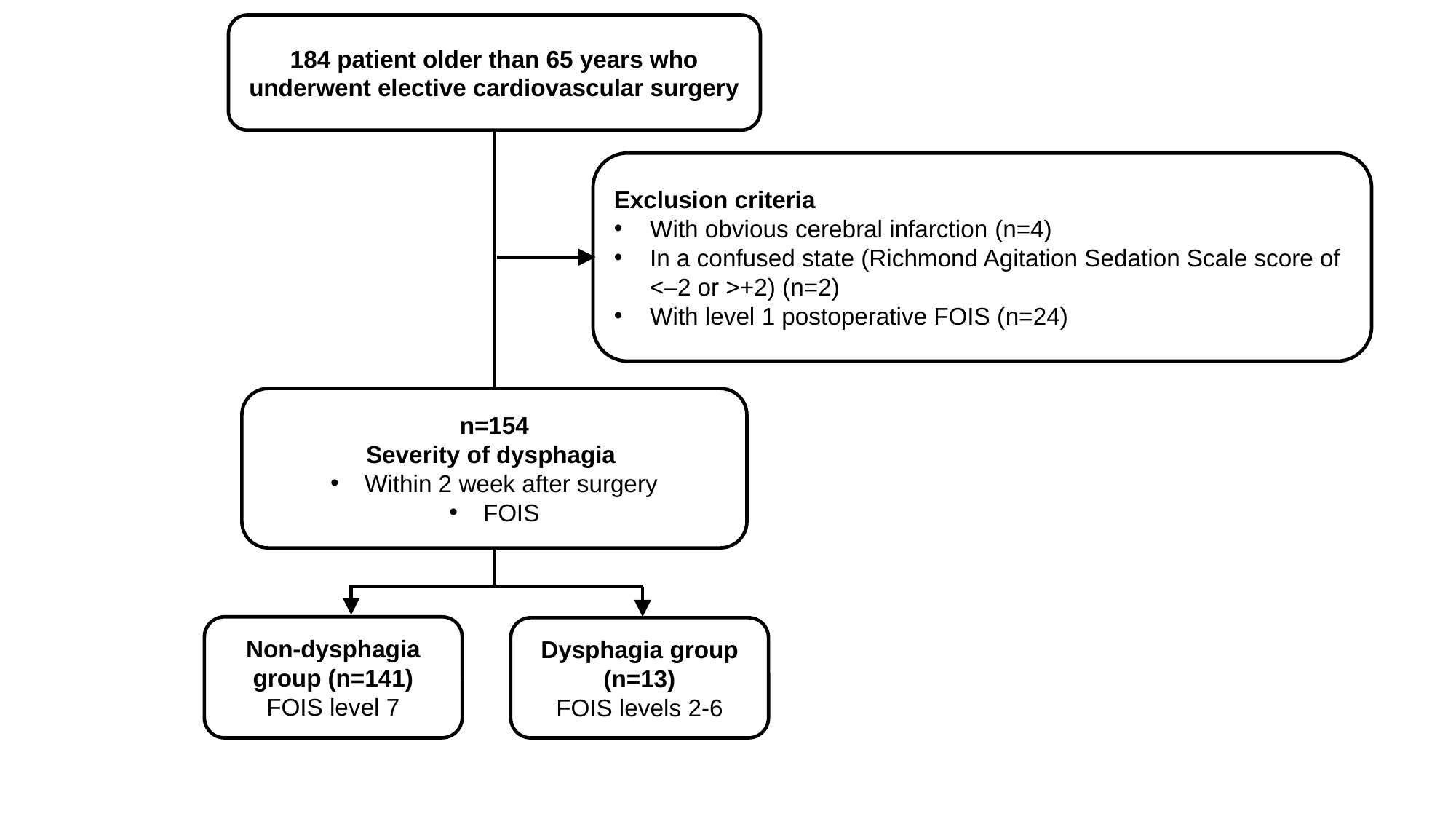

184 patient older than 65 years who underwent elective cardiovascular surgery
Exclusion criteria
With obvious cerebral infarction (n=4)
In a confused state (Richmond Agitation Sedation Scale score of <–2 or >+2) (n=2)
With level 1 postoperative FOIS (n=24)
n=154
Severity of dysphagia
Within 2 week after surgery
FOIS
Non-dysphagia group (n=141)
FOIS level 7
Dysphagia group (n=13)
FOIS levels 2-6
1
